# Cup‐like blasts in an adult patient with BCR‐ABL1 positive B‐cell acute lymphoblastic leukemia

**DOI:** 10.1002/jha2.532

**Published:** 2022-07-30

**Authors:** Zhenni Wang, Xing Jin, Jinlin Liu

**Affiliations:** ^1^ Laboratory Medicine Center, Department of Clinical Laboratory Zhejiang Provincial People's Hospital (Affiliated People's Hospital, Hangzhou Medical College) Hangzhou China; ^2^ Department of Hematology Zhejiang Provincial People's Hospital (Affiliated People's Hospital, Hangzhou Medical College) Hangzhou China

**Keywords:** ALL, acute leukemia, blood diseases

1

A 68‐year‐old female was presented to the hospital with complainment of asthenicus for 2 months. Initial hematological results showed leukocyte of 80.26 × 10^9^/L, hemoglobin of 134 g/L, and platelet of 401 × 10^9^/L. Peripheral blood smear revealed 80% blast cells with small or medium size, and 27% blast cells with frequent cup‐like nuclei (Figure 1A). Further bone marrow smear revealed hypercellular with 83% small‐ to medium‐sized blast cells, and 10% blast cells with cup‐like nuclei (Figure 1B). Myeloperoxidase (MPO) and nonspecific esterase were all negative. Further bone marrow immunophenotyping by flow cytometry were positive for CD34, CD19, HLA‐DR, CD22, CD10, CD58, and cyCD79a on blast cells, but other myeloid and T‐cell phenotype were negative. Then this patient was diagnosed with acute B‐cell lymphoblastic leukemia (B‐ALL). Furthermore, *BCR‐ABL1 p190* fusion gene was found in this patient, and 32 common lymphoid leukemia genes were sequenced and result showed the *IKZF1* mutation (exon5, c.4.88A>G, p.His163Arg).

**FIGURE 1:**
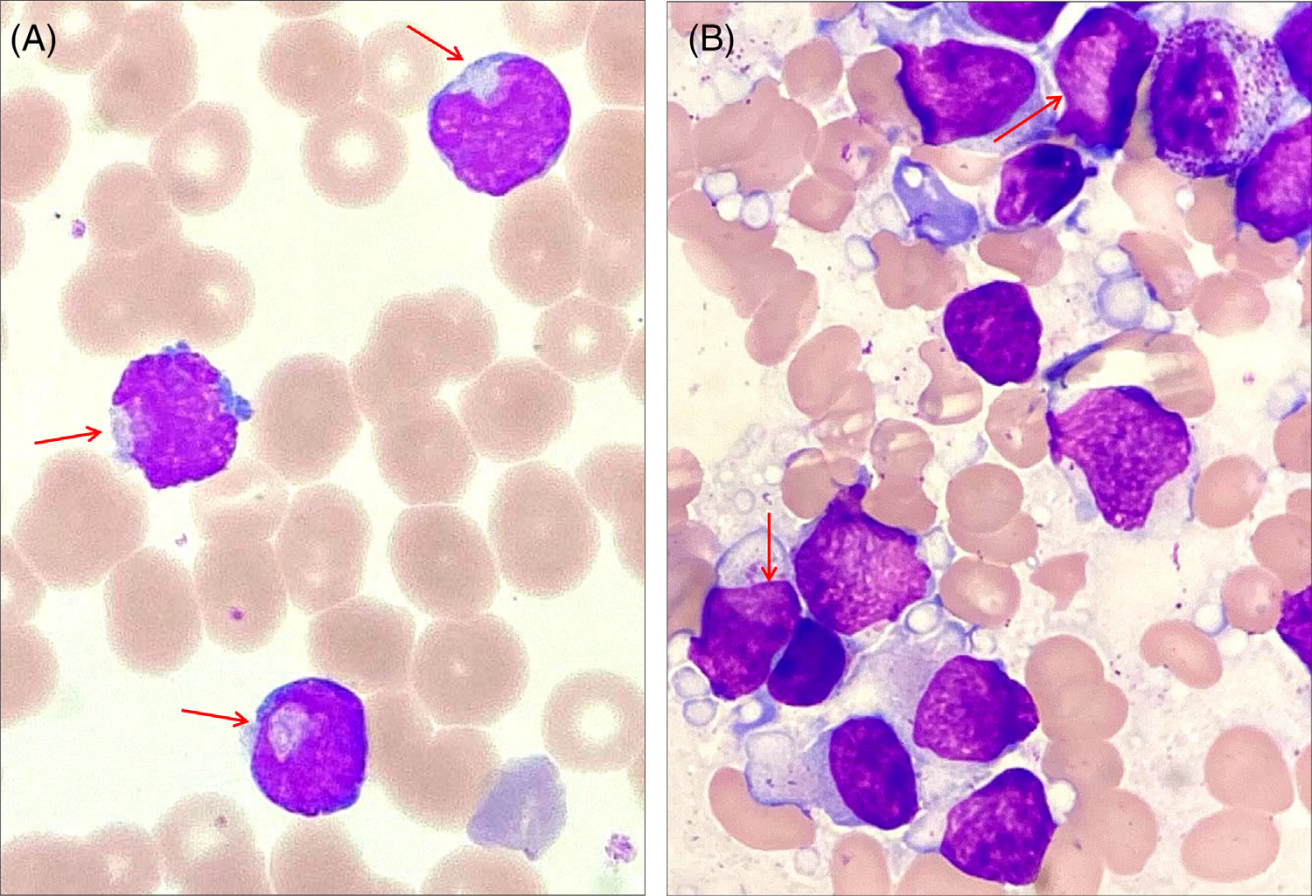
Cup‐like blasts in peripheral blood and bone marrow smear

Blasts with cup‐like morphology are well recognized in acute myeloid leukemia (AML), being frequently associated with a normal karyotype and mutated *NPM1* and/or *FLT3*‐*ITD*. Recently, a novel association between cup‐like nuclei and *IKZF1* deletion in pediatric B‐ALL had reported [1]. And these pediatric cup‐like nuclei cases are more likely to have an early B‐precursor phenotype, normal karyotype, or nonrecurrent cytogenetic abnormalities and are less likely to have favorable cytogenetic abnormalities [1]. Herein, we describe an unusual adult case of *BCR‐ABL1‐ p190* positive B‐ALL with cup‐like morphology. Whether this morphology of adult B‐ALL patient is related to the *BCR‐ABL1 p190* and/or *IKZF1* mutations needs further investigation.

## CONFLICT OF INTEREST

The authors declare that they have no conflict of interest.

## AUTHOR CONTRIBUTIONS

Zhenni Wang and Xing Jin provide the picture and clinical data. Jinlin Liu analyzed the data and wrote the manuscript.

## ETHICAL STATEMENT

This study has approved by the ethical committee of Zhejiang Provincial People's Hospital.

## PATIENT CONSENT STATEMENT

This study did not involve the personal information, only report the laboratory data. Then the patient consent was waived, and this waive was approved by the ethical committee of Zhejiang Provincial People's Hospital.

## FUNDING INFORMATION

The authors received no funding for this article.

## Data Availability

Data are available on request from the authors.
